# On Percussion

**Published:** 1843-01

**Authors:** John Hughes Bennett


*On Percussion.*—By John Hughes Bennett. Mediate percussion as employed by Piorry, is undoubtedly a more valuable means of diagnosis than is generally allowed. His experience, it is said, enables him to "map out, as it were, on the surface of the skin, with ink, the size and form of the heart, arch of the aorta, liver, spleen, kidney, &c.; and indicate to the eighth of an inch, the exact height of pleuritic effusion, or the margin of circumscribed pneumonic dulness." At first sight this partakes too much of the marvellous, but when we know the length of time he takes to percuss a single patient, (half an hour,) we may suppose him to be a more expert operator than the generality of practitioners. The instruments he uses are, 1st. *The Hammer:* the head of which is made of steel, brass, or iron; a capsule is screwed to the end with a projecting disc of caoutchouc; the handle is made of wood, with depressions for the fingers and thumb. The head of the handle is not placed exactly at right angles with the handle, but has a slight obliquity upwards. "This is necessary, because in employing it the handle is almost certain to be somewhat elevated, and this slight obliquity even then allows the practitioner to strike the pleximeter perpendicularly." 2d. *The Pleximeter:* made of ivory, wood, or metal, with a handle at each extremity, to enable the practitioner to take hold of it more readily.

*General rules to be followed in the practice of Mediate Percussion.*—1. The pleximeter should be held by the projecting handles between the thumb and index finger of the left hand, and pressed firmly down upon the organ to be percussed. Much depends upon this rule being followed, as the sound and sense of resistance are considerably modified according to the pressure made by the pleximeter. A very easy experiment will prove this. If, for instance, the pleximeter be struck while it rests lightly on the abdomen over the umbilicus, and again, when it is pressed firmly down amongst the viscera, the change in tone will be at once perceived. In the first case a sound is produced, from the muscles and integuments being alone influenced by the force of the blow; in the second case, a clear tympanitic sound is occasioned from the vibration of the walls of the intestine. In every instance, therefore, the pleximeter should be so held and pressed down, as to render it, so to speak, a part of the organ we wish to percuss.

2. Care must be taken to strike the pleximeter fairly and perpendicularly. Unless this be done, vibrations are communicated to textures in the neighborhood of the organ to be percussed, and fallacious results are the consequence. If, in percussing the lungs, for example, the blow be made obliquely, we obtain the dull sound produced by the rib, and I have seen considerable error in the diagnosis thus occasioned.

3. A strong or gentle stroke with the hammer will modify the tone and sense of resistance, inasmuch as the impulse may be communicated by one or the other to a deep-seated or a superficial organ. Thus a gentle stroke will elicit a pulmonal tympanitic sound just below the fourth rib, where a thin layer of lung covers the liver, but a strong one will cause a jecoral parenchymatous sound. At the inferior margin of the liver, on the other hand, where a thin layer of the organ covers the intestines, the reverse of this takes place, a gentle stroke occasioning a dull, and a strong one a clear sound.

4. By withdrawing the hammer immediately after the blow, we are better able to judge of the sound; by allowing it to remain a moment, we can judge better of the sense of resistance.

5. The integuments should not be stretched over the part percussed, as when the stethoscope is employed, for an unnatural degree of resistance is thus communicated to the hand of the operator from the muscular tension. In every case, especially where the abdomen is examined, the integuments and superficial muscles should be rendered as flaccid as possible.

6. It is always best to percuss on the naked skin. It is not absolutely essential, however, and in cases where, from motives of delicacy, it is desirable that the chest or abdomen be not exposed, it only becomes necessary that the covering of linen or flannel be of equal thickness throughout, and not thrown into folds.

*Special rules to be followed in percussing the chest.*—Percussion of the lungs generally bears reference to a change in density, which is only to be detected by comparing the healthy with the morbid portions. The great practical rule here to be followed, is to apply the pleximeter to both sides of the chest in succession, with the same firmness, exactly in the same situation, and let the blow with the hammer be given with the same force. Care must be taken that the position of both arms be alike, as the contraction of the pectoral muscles on one side more than on the other may induce error. In short, every circumstance must be the same before it is possible to determine in delicate cases, either from the tone or sense of resistance, whether change of density exist in the lungs. When circumscribed alterations are discovered in the pulmonary tissue, their limits may be marked out on the surface of the skin, in the manner previously indicated. In this way, I have frequently succeeded in determining with accuracy the size and form of circumscribed indurations, arising from partial pneumonia and pulmonary apoplexy. Under the clavicles, the pleximeter must be applied with great firmness. Inferiorly, a thin layer of lung lies over the superior surface of the liver; and to determine the exact place where its inferior border terminates, the blows with the hammer should be very slight. Posteriorly, also, the pleximeter must be firmly applied, and the force of the blows considerable: but they should decrease in force inferiorly, where a thin layer of lung descends over the liver much deeper than anteriorly.

In a healthy state, a distinct difference may be observed in the sonoriety of the lungs immediately after a full expiration and a full inspiration. This does not take place when the tissue becomes indurated from any cause; and thus we are furnished with a valuable diagnostic sign. Congestion of the lung, and pneumonia in its first stage, causes only slight dulness and increased resistance, which, however, are readily detected by the practised percussor. In the second and third stage of pneumonia, and in apoplexy of the lung, this dulness and resistance are well marked, and even an impression of hardness and solidity communicated to the hand. When, however, the lung is studded with tubercles, the induration is most intense, and the greatest degree of resistance communicated.

Partial induration from pneumonia, apoplexy, or tubercular deposition, may be detected by percussion, even when deep-seated and covered by healthy portions of the lungs. In this case, by pressing with the pleximeter, and striking lightly, a tympanitic sound is only heard; but by pressing the pleximeter down firmly, and striking with force, the dull sound may be elicited and circumscribed. When induration, however, exists inferiorly in those portions of the lungs which overlap the liver, it requires great practice to detect them with certainty. Caverns in the lungs, when large and filled with air, induce a tympanitic sound; but they are generally more or less full of viscous and fluid matters, and give rise to dulness.

Two or three ounces of fluid may be detected in the pleural cavity, by causing the patient to sit up. It is readily distinguished posteriorly, from the dulness of the liver on the right side; on the left, however, the limit between it and the spleen is not so well marked. Thc height or level of the fluid is readily determined, and should be marked daily by a line made with nitrate of silver. If the effusion be only on one side. the peculiar humoral dulness is more easily detected. It disappears on placing the patient in such a position as will cause the fluid to accumulate in another part of the pleural cavity, when the space, which was previously dull, becomes clear. When the effusion entirely fills the pleural cavity, no limit of course can be detected; but, even then, the dulness is distinguished from that of the liver by the diminished feeling of resistance.

When air is effused into the pleura, the sound is like that of a drum, and readily detected.—*Braithwaite's Retrospect.*

